# Optimising the Encapsulation of an Aqueous Bitter Melon Extract by Spray-Drying

**DOI:** 10.3390/foods4030400

**Published:** 2015-09-09

**Authors:** Sing Pei Tan, Tuyen Chan Kha, Sophie Parks, Costas Stathopoulos, Paul D. Roach

**Affiliations:** 1 School of Environmental and Life Sciences, University of Newcastle, New South Wales 2258, Australia; E-Mails: sophie.parks@dpi.nsw.gov.au (S.P.); paul.roach@newcastle.edu.au (P.R.); 2 Faculty of Food Science and Technology, Nong Lam University, Ho Chi Minh city, Vietnam; E-Mail: khachantuyen@hcmuaf.edu.vn; 3 Central Coast Primary Industries Centre, NSW Department of Primary Industries, New South Wales 2258, Australia; 4 Faculty of Bioscience Engineering, Ghent University Global Campus, Incheon 406-840, Korea; E-Mail: costas.stathopoulos@ghent.ac.kr

**Keywords:** bitter melon, encapsulation, maltodextrin, gum Arabic, spray drying, response surface methodology

## Abstract

Our aim was to optimise the encapsulation of an aqueous bitter melon extract by spray-drying with maltodextrin (MD) and gum Arabic (GA). The response surface methodology models accurately predicted the process yield and retentions of bioactive concentrations and activity (*R*^2^ > 0.87). The optimal formulation was predicted and validated as 35% (w/w) stock solution (MD:GA, 1:1) and a ratio of 1.5:1 g/g of the extract to the stock solution. The spray-dried powder had a high process yield (66.2% ± 9.4%) and high retention (>79.5% ± 8.4%) and the quality of the powder was high. Therefore, the bitter melon extract was well encapsulated into a powder using MD/GA and spray-drying.

## 1. Introduction

Bitter melon (*Momordica charantia* L.) is a tropical fruit that is considered useful in therapeutic ethnobotanical practice and Asian traditional medicine. Many medicinal properties, especially for the treatment of diabetes [1,2], have been proposed for various extracts of different parts of the bitter melon [2,3]. The pharmacological effects and plausible mechanisms of action of these extracts have been studied in animal models and cell culture and this body of work suggests that the fruit can have important health benefits [4,5]. Bitter melon has also been found to contain a wide range of bioactive compounds such as saponins, phenolics and flavonoids to which the proposed health benefits have been attributed [6,7,8].

We have previously optimised the aqueous extraction of saponins from bitter melon and used spray-drying to prepare a saponin-enriched bitter melon powder [9]. Spray-drying is one of the most popular methods available for preparing powders because it is easy to industrialise and allows for continuous production [10]. However, more than 80% of the saponins were not recovered after spray-drying [9]. This high loss may have been due to the high drying temperatures (150–220 °C) [11] needed for spray-drying, causing degradation of the saponins or to the saponins sticking to the wall of the chamber of the spray drier, which is a known issue during spray-drying [9]. The addition of encapsulating agents, such as maltodextrin, has been shown to have several positive effects during spray-drying including protecting bioactive compounds from degradation and reducing the loss of material on the wall of the spray-drying chamber by decreasing the stickiness of the material [12,13].

Apart from improving the drying process, encapsulation can prevent the absorption of moisture from the atmosphere and reduce agglomeration problems during storage and can improve the stability of the bioactive compounds during storage or during their incorporation into functional foods that require exposure to acid and/or alkaline solutions or high temperatures during their preparation.

In order to achieve a good retention of core material during spray-drying without compromising its quality, the product formulation needs to be optimised. Generally, a good encapsulating agent should possess several properties: low viscosity, non-hygroscopic, inherent film-forming capacity, bland in flavour/tasteless, non-reactive with the core material, soluble in aqueous solvents, inexpensive, food-grade, flexible, hard, thin and pliable [14]. However, no single encapsulating agent possesses all the above-mentioned properties, and therefore, two or more agents are often used in particular combinations.

Maltodextrin (MD) and gum Arabic (GA) are two of the most commonly used encapsulating agents for spray-drying due to their low viscosity and good solubility in aqueous solutions and they are often used in combination because they complement each other well [10,15].

The aim of this study was to maximise the encapsulation of the aqueous bitter melon extract with MD and GA using spray-drying by optimising the formulation of the solution to be spray-dried in terms of the concentrations of MD and GA relative to each other, the total concentration of both MD and GA in the encapsulating agents’ stock solution and the ratio of the aqueous bitter melon extract to the encapsulating agents’ stock solution in the infeed solution to be spray-dried. The effect of the different formulations on the process yield and efficiency after spray-drying was determined in order to maximise the retention of the bitter melon bioactive compounds and their antioxidant activity in the encapsulated powder produced. The quality of the encapsulated bitter melon powder prepared using the optimised formulation was then assessed by determining its moisture content, water activity, bulk density, water solubility and water absorption.

## 2. Materials and Methods

### 2.1. Chemicals

Folin-Ciocalteau (FC) reagent, sodium carbonate, sodium nitrite, aluminium chloride, sodium hydroxide, sodium acetate trihydrate, acetic acid, 2,4,6-tripyridyl-s-triazine (TPTZ), ferric (III) chloride hexahydrate, vanillin, sulphuric acid and assay standards, including trolox, gallic acid, rutin and aecsin, were purchased from Sigma (Castle Hill, NSW, Australia). Ethanol was purchased from Fronine (Taren Point, NSW, Australia). The encapsulating agents, MD (DE 18) and GA, were purchased from the Melbourne Food Depot (Brunswick East, Vic, Australia). Deionised water (DI) was prepared fresh before use using a Millipore Milli-Q water purification system (North Ryde, NSW, Australia).

### 2.2. Plant Materials

Fresh bitter melons (Moonlight variety) were purchased from the Sydney Markets (Sydney, NSW, Australia) and stored at −20 °C until used. Freeze-dried bitter melon powder was prepared by slicing the frozen bitter melons and placing the slices in liquid nitrogen for a few seconds before they were dried at −40 °C and 2 × 10^−1^ mbar for 72 h using a FD3 freeze dryer (Rietschle Thomas, Seven Hills, NSW, Australia). The freeze-dried samples were ground with a commercial blender (John Morris Scientific, Chatswood, NSW, Australia) and passed through a 1 mm EFL 2000 stainless steel sieve (Endecotts, London, England). The ground freeze-dried bitter melon preparation was then sealed and stored at −20 °C until used.

### 2.3. Preparation of the Aqueous Bitter Melon Extract

The aqueous bitter melon extract was prepared from the ground freeze-dried bitter melon according to the method described by Tan *et al.* [9] Briefly, 1g of the ground freeze-dried bitter melon was extracted with 20 mL of DI water at 40 °C for 15 min using a shaking water bath (Ratek Instruments, Boronia, VIC, Australia). The extraction mixture was then placed on ice for 10 min before it was vacuum-filtered, first through three layers of cheesecloth and then through a Whatman No. 1 filter paper (Lomb Scientific, Taren Point, NSW, Australia), to remove insoluble bitter melon residues. The extraction mixture was made fresh on the day to prepare the infeed solutions for spray-drying. The total solid content of the extracts was measured to be 2.04% ± 0.01% (w/w), as determined by weight difference after drying 10 g samples of the extracts at 80 °C for 24 h in a vacuum oven drier (Thermoline Scientific, Wetherill Park, NSW, Australia).

### 2.4. Experimental Design

First, the optimal ratio of MD to GA was determined using the single factor method. In this experiment, the overall concentration of the encapsulating agents in the stock solution was set at 30% (w/w) and the ratio of the aqueous bitter melon extract to the encapsulating agents’ stock solution was 1:1 (w/w). Several ratios of MD to GA, 1:0, 1:1, 3:2, 7:3 and 4:1 (w/w), were tested and the optimal ratio of MD to GA was determined based on the highest values for the process yield and the retentions of total saponin content (TSC), total phenolic content (TPC), total flavonoid content (TFC) and total antioxidant activity (TAA) for the encapsulated powder and this ratio was used in the subsequent experiments.

The RSM with central composite design (CCD) was then employed to determine the optimal overall concentration of the encapsulating agents (MD/GA, 1:1, w/w) in the stock solution and the optimal ratio of the aqueous bitter melon extract to the encapsulating agents’ stock solution in order to achieve the maximal process yield (Y_1_) and the highest retentions of TSC (Y_2_), TPC (Y_3_), TFC (Y_4_) and TAA (Y_5_). The experiment consisted of 11 runs with three central points. The levels for the independent variables (Table 1), which were the concentration of the encapsulating agents in the stock solution (X_1_) and the ratio of the aqueous bitter melon extract to the encapsulating agents’ stock solution (X_2_), were chosen based on several preliminary trials.

**Table 1:** The coded and uncoded levels for the two independent variables.

Coded Variable Levels	X_1_ Stock Solution Concentration % (w/w)	X_2_ Ratio of Extract to Stock Solution g/g (WW)
+1.682	37	1.71
+1	35	1.50
0	30	1.00
−1	25	0.50
−1.682	23	0.30

X_1_: The overall concentration of the encapsulation agents’ stock solution; X_2_: The ratio of the bitter melon extract to the encapsulation agents’ stock solution.

To express the process yield (Y_1_) and the retentions of TSC (Y_2_), TPC (Y_3_), TFC (Y_4_), and TAA (Y_5_) as a function of the independent variables, a second-order polynomial equation was generated as follows:

Y_i_ = a_0_ + a_1_X_1_ + a_2_X_2_ + a_11_X_12_ + a_22_X_22_ + a_12_X_1_X_2_ (1)

where Y is the dependent response; X_1_ and X_2_ are the levels of the independent variables; and a_0_, a_i_, a_ii_ and a_ij_ are the regression coefficients of the variables for the offset, linear, quadratic and interaction terms, respectively.

### 2.5. Preparation of Spray Drying Infeed Solutions

Stock solutions with the desired concentrations (Table 1) of the encapsulating agents (MD/GA, 1;1, w/w) were prepared in DI water using a Silverson L4RT high shear mixer (J L Lennard Pty. Ltd., Silverwater, NSW, Australia) at 4500 rpm for 10 min. Then, different amounts of the aqueous bitter melon extract were added directly to the encapsulating agent stock solution to give the desired ratios of the bitter melon extract to the encapsulation agents (Table 1) on a wet weight basis (WW). The mixtures were homogenised with the Silverson L4RT mixer for 10 min and then placed in an ultrasound water bath (Ultrasonik 57X, Extech Equipment Pty. Ltd., Wantirna South, VIC, Australia) for 20 min to promote the microencapsulation process [16].

### 2.6. Spray-Drying Conditions

The spray-drying process was conducted using a laboratory-scale spray drier (Buchi Mini Spray Drier B-290, Noble Park, VIC, Australia) according to a method described by Fang and Bhandari [17] with slight modifications. For each of the infeed solutions, 300 mL was sprayed through a 0.7 mm diameter nozzle tip by the co-current flow atomiser into the in drying chamber the same direction as the drying air flow. The drying air flow rate, the compressed air flow rate and the feed rate were set at 35 m^3^/h, 473 L/h and 14–16 mL/min, respectively. The inlet temperature was set at 150 ± 2 °C, the outlet temperature at 85 ± 2 °C and the aspiration at 100%. The outlet temperature was controlled by the flow rate. After spray-drying, the encapsulated powder was collected from the glass container after the container was cooled in a desiccator containing silica gel to prevent moisture absorption and weighed for powder yield determination. The encapsulated powder was then transferred to vacuum-sealed plastic bags (The Packaging Centre Pty Ltd, Wattle Glen, VIC, Australia) and stored at −20°C until used.

All experimental runs to prepare the encapsulated powders were done in triplicate. The measurements on each solution prepared for spray-drying and of the encapsulated powder were conducted in triplicate and the mean was used as a value in the subsequent statistical analysis for each set of three experimental runs.

### 2.7. Analytical Methods

#### 2.7.1. Measurement of Bioactive Compounds

The concentration of bioactive compounds was determined in the aqueous bitter melon extracts and in the spray dried encapsulated powders. However, before their analysis, the bioactive compounds first needed to be extracted from the encapsulated powders. The appropriate amount of each encapsulated powder was dissolved in 15 mL of DI water to give the reconstituted powder sample the same total solid content as the solution used to prepare the powder by spray-drying (the parent infeed solution). For the extraction, 20 mL of ethanol was added to 10 mL of the parent solutions or the reconstituted powders and the samples were incubated at 50 °C for 15 min in a shaking water bath. After extraction, the samples were cooled on ice for 10 min and then centrifuged at 4350× *g* for 10 min at 10 °C in a Beckman JA-20 rotor and a J20 MC Centrifuge (Beckman Instruments Inc., Palo Alto, CA, USA). The supernatant from each ethanolic extract was filtered through a 0.45 μm syringe filter (Phenomenex, Pennants Hills, NSW, Australia) and used for the analyses of the bioactive compounds.

The TSC was measured by a method described by Hiai, *et al.* [18] with some modifications. Each extract (0.3 mL of aqueous bitter melon extract or 0.3 mL of the encapsulated powder ethanolic extract) was mixed with 0.3 mL of 8% (*w*/*v*) vanillin solution and 3 mL of 72% (*v*/*v*) sulphuric acid. The mixture was mixed and incubated at 60 °C for 15 min and then cooled on ice for 10 min. The absorbance of the mixture was measured at 560 nm using a spectrophotometer (Carry 50 Bio, Varian Pty. Ltd., Mulgrave, VIC, Australia). Aecsin was used as a standard and the results were expressed as mg aecsin equivalents (AE) per g of dry weight (DW) of the sample (mg AE/g).

The method of Cicco, *et al.* [19] was used to determine the TPC with slight modifications. Briefly, 0.3 mL of each extract was mixed with 0.3 mL of the FC reagent. The solution was mixed well and incubated at room temperature (RT) for 2 min to equilibrate. Then, 2.4 mL of a 5% (*w*/*v*) sodium carbonate solution was added and the solution mixed and incubated at RT for 2 h. The absorbance of the solution was recorded at 765 nm using the spectrophotometer. Gallic acid was used as a standard and the results were expressed as mg gallic acid equivalents (GAE) per g DW of the sample (mg GAE/g).

The TFC was determined according to a method described by Wu and Ng [20] with some modifications. Briefly, 0.5 mL of each extract was mixed with 2 mL of DI water followed by the addition of 0.15 mL of 5% (*w*/*v*) sodium nitrite solution. After 6 min, 0.15 mL of 10% (*w*/*v*) aluminum chloride was added and incubated at RT for another 6 min. Then, 2 mL of 4% (*w*/*v*) sodium hydroxide was added. The solution was then made up to 5 mL with DI water, mixed and placed in the dark at RT for 15 min. The absorbance of the solution was measured at 510 nm using the spectrophotometer. Rutin was used as a standard and the results were expressed as mg rutin equivalents (RE) per g DW of the sample (mg RE/g).

#### 2.7.2. Measurement of Antioxidant Capacity

The TAA was determined using the ferric reducing antioxidant power (FRAP) assay as described by Benzie and Strain [21] with some modifications. The stock solutions of 300 mM acetate buffer (pH 3.6), 10 mM TPTZ in 40 mMHCl and 20 mM ferric (III) chloride hexahydrate in DI water were prepared and kept at 4 °C until used. A fresh working solution was prepared by mixing 100 mL of acetate buffer, 10 mL of TPTZ and 10 mL of ferric (III) chloride hexahydrate in a ratio of 10:1:1 and incubated at 37 °C for 30 min before used. Briefly, 0.15 mL of each extract was mixed with 2.85 mL of the working solution for 30 min in the dark at RT. The absorbance of the solution at 593 nm was measured using the spectrophotometer against a reagent blank. Trolox was used as a standard and the results were expressed as μmole trolox equivalents (TE) per g DW of the sample (μmole TE/g).

#### 2.7.3. Determinants of the Infeed Solutions

The total solid content of the infeed solutions was determined by weight difference after drying the solutions at 80 °C for 24 h in a vacuum oven drier (Thermoline Scientific, Wetherill Park, NSW, Australia).

The stability of the infeed solutions was determined with a COULTER QuickSCAN (Coulter Corporation, Miami, FL, USA) using the delta back-scattering technique. Briefly, 10 mL of each solution was scanned every hour for 24 h at the ambient temperature. The device scanned the sample automatically and converted the macroscopic profile of the mixture into a graphic. In order to observe variations in the profile more easily, the curve obtained after the first hour was set as a reference profile and it was subtracted from all the other profiles (2 to 24 h). The results were expressed as % delta back-scattering.

#### 2.7.4. Determinants of the Encapsulated Powders

##### Calculation of Process Yield

The process yield was calculated by dividing the dry weight of the encapsulated powder recovered in the glass collection container after spray-drying by the dry weight of the material (total solid content) in the infeed solution, which was spray dried, and expressed as a percentage.

##### Retentions of Bioactive Concentration and Activity

The retention was calculated and expressed as the percentage of the TSC, TPC, TFC and TAA concentration (mg/g DW) in the infeed solution, which was recovered in the encapsulated powder after spray-drying as follows:

(2) $$Retention\;of\;bioactive\;concentration\;or\;activity\;\left(\%\right)=\left(\frac{bioactive\;concentration\;or\;activity\;in\;encapsulated\;powder}{bioactive\;concentration\;or\;activity\;in\;infeed\;solution}\right)\times 100\%$$

##### Determination of the Physical Properties

The moisture content was determined by weight difference after drying the powders at 80 °C for 24 h in a vacuum oven drier (Thermoline Scientific, Wetherill Park, NSW, Australia). Water activity was measured using a water activity meter (AquaLabPawkit, Pullman, WA, USA). To determine the bulk density, 2 g of each encapsulated powder was placed into a 10 mL measuring cylinder and homogenised using a vortex mixer (Ratek Instrument, Boronia, VIC, Australia). The vortex was stopped after 1 min and powder was allowed to settle down freely. This volume of the settled powder was used to determine its bulk density and it was calculated by dividing the weight of the powder by the volume occupied in the cylinder (g/mL).

The water solubility index (WSI) and the water absorption index (WAI) of the powders were determined using the reconstitution method of Anderson *et al.* [22] with some modifications. Each powder (2.5 g) was dispersed in 25 mL of DI water and vigorously agitated using a vortex mixer for 30 min at RT. The solution was transferred to a centrifuge tube and centrifuged at 4350× *g* for 10 min in a Beckman JA-20 rotor and a J20 MC Centrifuge (Beckman Instruments Inc., Palo Alto, CA, USA). Finally, the supernatant and the precipitate were separated and dried at 80 °C for 24 h in a vacuum oven drier (Thermoline Scientific, Wetherill Park, NSW, Australia). The WSI was calculated as the difference between the weight of the powder obtained after drying the supernatant and the weight of powder used in the reconstitution test, and expressed as a percentage. The WAI was calculated as the difference between the weight of the material obtained after drying the precipitate and the weight of powder used in the reconstitution test, and expressed as a percentage.

To measure the colour of the encapsulated powder prepared using the optimal formulation, the powder was packed into a polyethylene pouch and measured using a Minolta chroma meter (CR-400 Chroma meter, Konica Minolta Sensing, Osaka, Japan). The meter was calibrated with a white standard tile before measurements were done. The samples were packed into a polyethylene pouch for measurement and the results were expressed as Hunter colour values of L, a, and b and the Chroma (C) was calculated by the formula (a^2^ + b^2^)^1/2^ and the hue angle (H°) was calculated by the formula: arctan (b/a).

##### Scanning Electron Microscope (SEM)

The field-emission SEM (Hitachi S-4800, Ibaraki, Japan) was operated at a voltage of 5 kV to determine the particle morphology and size of the encapsulated powder. A small amount of the encapsulated powder was fixed onto the aluminium specimen holder with double-sided tape. The specimen holder was placed in an Emitech K550 sputter coater (Emitech, Ashford, UK) under vacuum and the powders were coated with a fine layer of gold palladium. The coated samples were then observed under the SEM at 5000 and 10,000 × magnifications [23].

### 2.8. Statistical Analyses

The second-order polynomial models and the three-dimensional (3D) surface and two-dimensional (2D) contour plots for the process yield and the retentions of TSC, TPC, TFC and TAA were generated using the JMP software version 11.0 (SAS Institute Inc., Cary, NC, USA). The adequacy of the models was determined based on the coefficient of determination (*R*^2^) and the lack of fit. Results were expressed as mean values with standard deviations. Significant differences between means were determined by analysis of variance (ANOVA), the Bonferroni post-hoc test and the 5% significance level (*p* < 0.05) using the SPSS software version 19.0 (IBM Australia Limited, St. Leonard, NSW, Australia). Pearson correlation coefficients were also determined using the SPSS software.

## 3. Results and Discussion

### 3.1. The Maltodextrin to Gum Arabic Ratio

With the overall concentration of the encapsulating agents in the stock solution set at 30% and the ratio of the extract to the encapsulating agents’ solution set at 1:1 (w/w), several ratios of MD to GA in the stock solution, 1:0, 1:1, 3:2, 7:3 and 4:1 (w/w), were tested for their effects on the process yield and retentions of bioactive concentrations and activity (Table 2). The ratio of the two encapsulating agents had no effect on the process yield. However, it did have an effect on the retentions of TSC, TPC, TFC and TAA and the optimal MD to GA ratio was determined to be 1:1 (w/w) because it consistently resulted in the highest values for all four retentions, especially for TAA (Table 2), and this ratio was used in the subsequent experiments.

**Table 2:** The effect of different ratios of maltodextrin (MD) to gum Arabic (GA) on the encapsulation yield (EY) and the retentions of total saponin content (TSC), total phenolic content (TPC), total flavonoid content (TFC) and total activity activity (TAA) of the encapsulated powder.

Ratio of MD to GA g/g (WW)	EY (%)	TSC (%)	TPC (%)	TFC (%)	TAA (%)
1:0	51.29 ± 4.52 ^a^	41.70 ± 0.95 ^a^	42.60 ± 0.12 ^a^	20.50 ± 0.40 ^a^	25.25 ± 1.35 ^a^
1:1	61.97 ± 4.24 ^a^	61.58 ± 1.33 ^bc^	57.85 ± 1.25 ^bc^	50.79 ± 1.12 ^b^	47.16 ± 2.78 ^b^
3:2	56.82 ± 3.06 ^a^	64.00 ± 1.39 ^b^	59.66 ± 0.73 ^b^	51.14 ± 1.28 ^b^	40.95 ± 1.75 ^c^
7:3	62.15 ± 4.32 ^a^	61.57 ± 1.27 ^c^	59.04 ± 1.39 ^b^	43.84 ± 0.77 ^c^	35.84 ± 2.41 ^c^
4:1	57.63 ± 4.63 ^a^	60.29 ± 0.44 ^c^	55.41 ± 0.30 ^c^	36.92 ± 0.78 ^d^	28.40 ± 0.71 ^a^

Values in a column not sharing a superscript letter (^a, b, c, d^) are significantly different from each other (*p* < 0.05).

### 3.2. Fitting the Response Surface Methodology Model

The RSM with Central Composite Design (CCD) was then employed to determine the optimal formulation for the infeed formula in terms of the overall concentration of the encapsulating agents (MD/GA, 1:1, w/w) in the stock solution and the optimal ratio of the aqueous bitter melon extract to the encapsulating agents’ stock solution (Table 3), using the concentrations (X_1_) and the ratios (X_2_) listed in Table 1. The combinations generated by the CCD model for X_1_ and X_2_ and carried out experimentally are shown in Table 3. The experimental data (Table 3) for the process yield and the retentions of TSC, TPC, TFC and TAA were analysed using multiple regression and response surface analysis to generate the final equations in terms of the two independent variables, concentration (X_1_) and ratio (X_2_). The predicted data (Table 3) for all responses were calculated using the equations generated by the JMP software.

Table 4 shows the regression coefficients of the process yield with the independent variables (concentration and ratio). As determined using the P-value, the process yield coefficients were significant for first-order linear effects, with the concentration directly related and the ratio inversely related. However, the values for the second-order quadratic and interactive effects were not significant (Table 4).

The coefficients of the retentions of TSC, TPC, TFC and TAA were also significant for the first-order linear effects, with both the concentration and the ratio being directly related (Table 4). However, except for the retentions of TSC, the coefficients of the retentions were not significant for the linear effect of the ratio (Table 4). In contrast to the process yield, the coefficients of the retentions were all significant for the second-order quadratic effects, with both the concentration and the ratio directly related (Table 4). The second-order interactive coefficients of the retentions of TSC, TPC, TFC and TAA were not significant (Table 4).

To generate the models, only the significant regression coefficients (Table 4) were taken into account. The final equations in terms of the two independent variables, concentrations (X_1_) and ratios (X_2_) for process yield, and retentions of TSC, TPC, TFC and TAA to obtain the predicted values in Table 3 were as follows:

Process yield = 99.34 − 0.76 X_1_ − 66.56 X_2_ + 1.78 X_1_ X_2_ (3)

Retention of TSC = 217.79 − 10.56 X_1_ − 26.57 X_2_ + 0.19 X_12_ + 18.10 X_22_ (4)

Retention of TPC = 520.25 − 28.18 X_1_ − 135.48 X_2_ + 0.50 X_12_ + 66.67 X_22_ (5)

Retention of TFC = 334.26 − 19.93 X_1_ − 56.10 X_2_ + 0.38 X_12_ + 27.23 X_22_ (6)

Retention of TAA = 596.13 − 32.25 X_1_ − 178.44 X_2_ + 0.56 X_12_ + 89.92 X_22_ (7)

**Table 3:** The experimental (Exp.) and predicted (Pred.) values for the encapsulation yield (EY) and the retentions of total saponin (TSC), total phenolic (TPC) and total flavonoid (TFC) content and the total antioxidant activity (TAA) for the bitter melon extract encapsulated powder obtained from the RSM CCD design.

Pattern *	X_1_ Concentration % (w/w)	X_2_ Ratio g/g (WW)	EY (%)		TSC (%)		TPC (%)		TFC (%)		TAA (%)	
			Exp.	Pred.	Exp.	Pred.	Exp.	Pred.	Exp.	Pred.	Exp.	Pred.
00	30	1.00	63.47	63.38	60.10	63.52	54.98	56.04	48.68	49.49	45.79	44.11
00	30	1.00	65.89	63.38	62.67	63.52	57.28	56.04	51.16	49.49	43.54	44.11
0A	30	1.71	52.15	54.17	74.23	79.13	87.81	87.21	62.95	61.68	90.02	89.15
+−	35	0.50	70.78	70.61	71.49	72.18	95.50	95.38	81.08	80.97	87.70	86.64
0a	30	0.30	72.81	72.59	63.07	65.65	91.61	90.21	63.37	63.98	87.48	87.19
00	30	1.00	62.54	63.38	61.98	63.52	55.28	56.04	51.34	49.49	43.66	44.11
++	35	1.50	68.21	66.35	79.97	81.81	90.18	93.24	78.35	79.33	83.31	88.04
A0	37	1.00	70.50	70.62	79.92	79.06	97.84	94.17	92.91	89.02	85.27	81.92
−+	25	1.50	43.15	47.25	77.47	73.41	80.19	75.04	53.82	50.63	80.37	74.54
a0	23	1.00	64.46	56.14	59.06	67.13	63.95	68.32	44.28	48.27	58.35	62.75
−−	25	0.50	63.56	69.31	63.20	63.78	79.46	77.18	57.07	52.27	74.01	73.14

*: Experiments were conducted in random order; **X_1_**: The overall concentration of the encapsulation agents’ stock solution; **X_2_**: The ratio of the bitter melon extract to the encapsulation agents’ stock solution.

**Table 4:** The coded second-order regression coefficients for the encapsulation yield (EY) and the retentions of total saponin (TSC), total phenolic (TPC) and total flavonoid (TFC) content and the total antioxidant activity (TAA) for the bitter melon extract encapsulated powder obtained from the RSM CCD design.

Independent Variables	Regression Coefficient Values				
	EY (Y_1_)	TSC (Y_2_)	TPC(Y_3_)	TFC (Y_4_)	TAA (Y_5_)
Intercept	63.97	61.58	56.51	50.39	44.33
Linear					
X_1_	5.10 *	5.04 *	9.24 **	14.66 ***	6.84 *
X_2_	−6.53 *	4.82 *	−1.07	−0.82	0.70
Quadratic					
X_1_ X_1_	0.87	4.94	12.51 **	9.53 *	14.01 **
X_2_ X_2_	−1.63	4.53 *	16.67 **	6.81 *	22.48 ***
Interaction					
X_1_X_2_	4.46	−1.45	−1.51	0.13	−2.69
R^2^	0.86	0.87	0.98	0.97	0.98
*p*-value of lack of fit	0.083	0.062	0.071	0.100	0.075

X_1_: The overall concentration of the encapsulation agents’ stock solution; X_2_: The ratio of the bitter melon extract to the encapsulation agents’ stock solution; *: *p* < 0.05; **: *p* < 0.01; ***: *p* < 0.0001.

Table 4 also shows that the predicted data from the equations and experimental data were highly correlated; the coefficient of multiple determination (*R*^2^) for the process yield (*R*^2^ = 0.86) and the retentions of TSC (*R*^2^ = 0.87), TPC (*R*^2^ = 0.98), TFC (*R*^2^ = 0.97) and TAA (*R*^2^ = 0.98) were all very high. In addition, the lack of fit for the process yield and the retentions of TSC, TPC, TFC and TAA were found to be non-significant (*p* > 0.05) (Table 4). This indicated that the second-order polynomial models (Equations (3)–(7)) were adequate to describe the true behaviour of the system and could be used for interpolating the experimental data.

### 3.3. Effects of the Concentration and Ratio on Encapsulation Yield and Encapsulation Efficiency

It is important to obtain as high a process yield as possible from any encapsulation process, including spray-drying. If the yield is low, the encapsulation may not be economical and therefore not worth doing. However, in order to maximise the yield, it is important that the effects of the important parameters of the system are known. For spray-drying, two of the parameters, which can be varied in the infeed solution, are the concentration of the encapsulating agents in the stock solution (X_1_) and the ratio of the extract solution to the encapsulating agents’ solution (X_2_).

**Figure 1:**
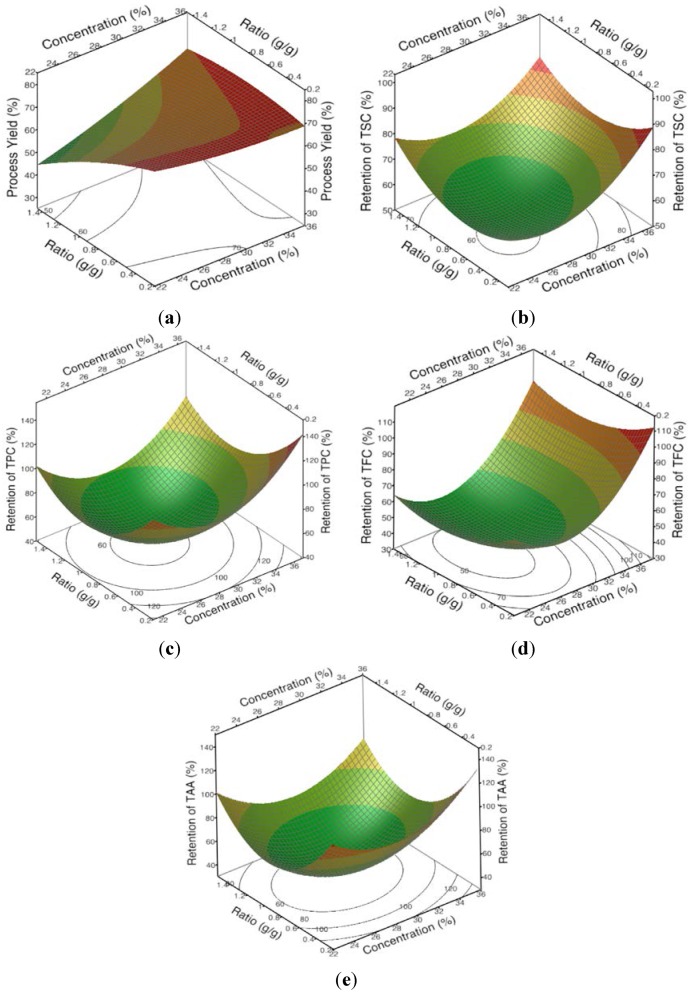
The 3D response surface and 2D contour plots of the (**a**) process yield and the retentions of (**b**) total saponin content (TSC); (**c**) total phenolic content (TPC); (**d**) total flavonoid content (TFC) and (**e**) total antioxidant activity (TAA) in response to concentration (X_1_) and ratio (X_2_).

In the present study, Table 4 and the 3D response surface and 2D contour plot (Figure 1a) show that the process yield significantly increased as the concentration (X_1_) of the encapsulating agents increased and the ratio of the extract to the encapsulating agent (X_2_) decreased. The yield of encapsulated powder from the spray-drying process is mainly determined by the efficiency with which the powder is collected. Usually, a low process yield is due to the sprayed droplets and powder sticking to the wall of the chamber and cyclone before they are sufficiently dry and therefore, this material is unable to be collected [24,25,26].

The lower yields obtained from spray-drying with the lower concentrations of the encapsulating agents in the stock solution (Figure 1a, Table 4, Equation (3)) and the higher ratios of the extract solution to the encapsulating agent solution are both likely to have been caused by an insufficient amount of the encapsulating agents being available to completely cover the sprayed water droplets, which would have caused some of the droplets to stick to the spray dryer’s chamber wall before they were sufficiently dry [25]. Essentially, the lower concentrations of the encapsulating agents in the agents’ aqueous stock solution and adding relatively more of the aqueous bitter melon extract relative to the encapsulating agent stock solution (Table 1) both resulted in lower concentrations of the encapsulating agents in these infeed solutions.

The response for the retentions of TSC, TPC, TFC and TAA was more complicated than for the process yield. The retentions also generally increased as the concentration of the encapsulating agents was increased in the stock solution (Figure 1b–e, Table 4, Equations (4)–(7)) but it was more curvilinear than for the process yield. Furthermore, the ratio of the aqueous bitter melon extract to the encapsulating agents’ stock solution did not have a significant effect on the retentions of TPC, TFC and TAA (Table 4). There was a significant effect on the retention of TSC, but the effect was a direct effect, not an inverse effect as seen for the process yield (Table 4). Therefore, unlike the effects on the process yield, which appeared to be mainly due to the concentration of the encapsulating agents in the infeed solution, it was not apparent what the underlying reason was for the effects on the retentions.

**Figure 2:**
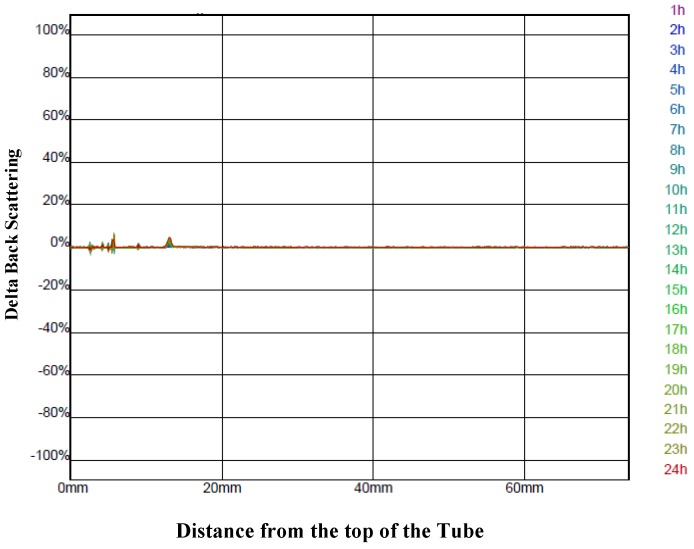
The delta back scattering profile measured every hour over 24 h for an infeed solution prepared with the encapsulating agents’ stock solution at 35% (w/w) and a ratio of 1.5:1 g/g of the aqueous bitter melon extract to the encapsulating agent solution. The graph is typical of the delta back scattering profile for all the other infeed solutions prepared with different concentrations and ratios.

Of note is the fact that none of the variations seen in the process yield and retentions were due to differences in the stability of the different infeed solutions. Measurement of the backscattering profiles (Figure 2) for all the infeed solutions showed that they were all very stable over 24 h and, therefore, the stability of the infeed solutions was unlikely to be a factor influencing their spray-drying.

### 3.4. The Optimal Formulation and Validation of the Models

**Figure 3:**
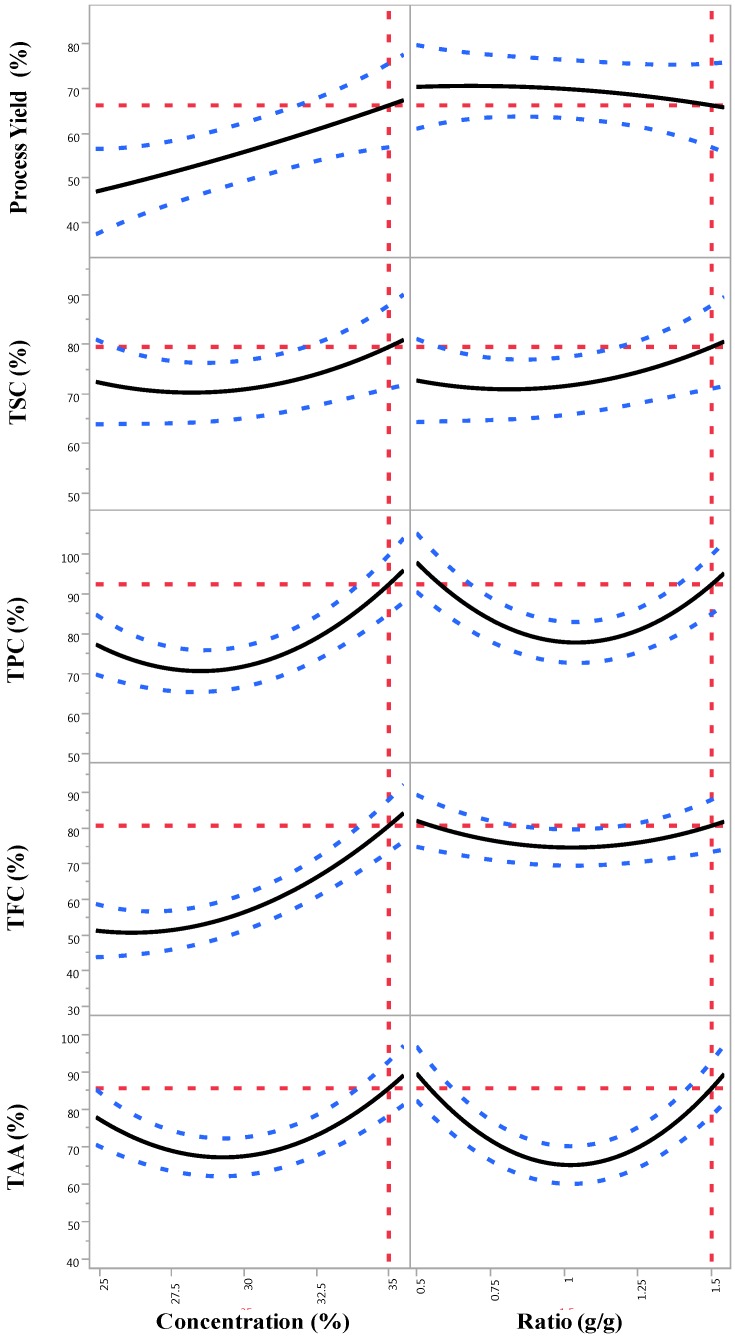
Prediction profiler plots for the process yield and the retentions of the process yield and the retentions of total saponin content (TSC), total phenolic content (TPC), total flavonoid content (TFC) and total antioxidant activity (TAA) of the encapsulated powder affected by concentration (X_1_) and ratio (X_2_).

The optimal formulation for obtaining the overall highest values for the process yield and the retentions of the bioactive compounds and antioxidant activity was predicted by the generated model shown in Figure 3. The theoretical maximum value for the optimal formulation was predicted to be a concentration (X_1_) of 35% (w/w) for the encapsulating agents’ stock solution and a ratio (X_2_) of 1.5:1 g/g of the aqueous bitter melon extract to the encapsulating agent solution.

The maximum response values predicted by the optimal formulation (X_1_ = 35%, X_2_ = 1.5:1 g/g) for the process yield and the retentions of TSC, TPC, TFC and TAA are shown in Figure 3 and Table 5. A verification experiment was performed in triplicate using the predicted optimal formulation to determine its validity. The mean values for the responses obtained from the real experiment were then compared with the predicted values. The results (Table 5) showed that there were no significant differences (*p* > 0.05) between the measured and the predicted values for the process yield and the retentions of TSC, TPC, TFC and TAA. Therefore, it was evident that the models (Equations (3)–(7)) were valid and reliable for predicting the optimal formulation.

**Table 5:** Validation of the models. The predicted and experimental values for the encapsulation yield (EY) and the retentions of the total saponin (TSC), total phenolic (TPC) and total flavonoid (TFC) content and the total antioxidant activity (TAA) for the bitter melon extract encapsulated powder obtained for the optimal conditions.

	EY (Y_1_)	TSC (Y_2_)	TPC (Y_3_)	TFC (Y_4_)	TAA (Y_5_)
Predicted values	66.4 ± 4.4	80.9 ± 3.9	93.9 ± 3.5	80.6 ± 3.3	88.3 ± 3.9
Experimental values	62.4 ± 0.4	82.7 ± 1.6	92.0 ± 3.6	79.6 ± 3.5	83.6 ± 3.9

According to these results, a stock solution concentration of 35% (w/w) for the encapsulating agents appeared to be optimal for effectively forming a protective covering over the surface of the droplets, containing the bitter melon extract, during the spray drying process and thus to result in a high process yield and high retentions [27]. Such a high encapsulating agent concentration is thought to result in a dense and tightly packed continuous phase membrane which can efficiently surround dispersed solutes, such as the saponins, phenolics and flavonoids from the aqueous bitter melon extract in the present study, and thereby prevent the degradation of these bioactive compounds due to exposure to heat and oxidation [28]. The antioxidant activity of such solutes can also be preserved, and as seen in another study [15], the TSC, TPC and TFC of the encapsulated bitter melon extract were highly correlated with the TAA; the correlation coefficients were 0.71, 0.97 and 0.68, respectively.

### 3.5. Properties of the Optimised Encapsulated Powder

**Figure 4:**
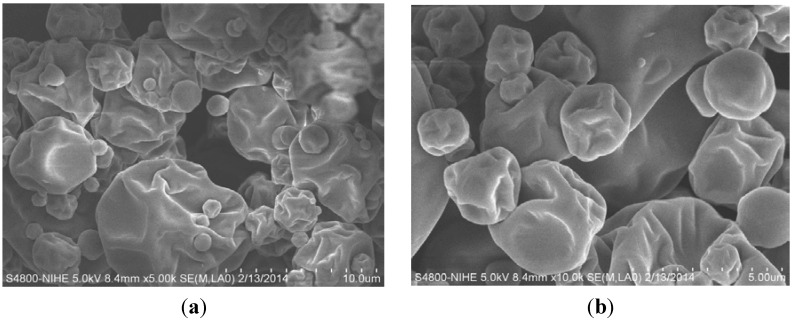
Outer microstructure of the encapsulated powder prepared with the optimal infeed solution formulation, 35% (w/w) maltodextrin and gum arabic (MD/GA, 1:1) with a ratio of aqueous bitter melon extract to MD:GA of 1.5:1 g/g at magnifications of (**a**) ×5000 and (**b**) ×10,000.

The SEM images of the encapsulated powder prepared with the optimal formulation (X_1_ = 35%, X_2_ = 1.5:1 g/g) are shown in Figure 4. Spherical particles of different sizes were observed, a well-known characteristic of spray-dried powders [25]. The powder particles were micron-sized (<12 μm). As seen in Figure 4, the encapsulated powders were free of cracks and pores, which is very important to prevent the bitter melon extract from being exposed to the atmosphere and being degraded through oxidation.

However, the SEM images also showed that the surface of the powder particles was wrinkled, which may have been due to the spray drying temperatures used [29]. According to Nijdam and Langrish [30], a vacuole forms once a skin has developed on the surface of a droplet, and it inflates when the temperature and the vapour pressure inside the vacuole are higher than the local ambient boiling point and pressure, respectively.

When the drying temperature is high, a large number of the powdered particles tend to have smooth surfaces because the moisture evaporates rapidly from the surface and the skin quickly becomes dry and rigid and keeps its smooth surface [30]. However, when the drying temperature is relatively low, the particles produced tend to shrink and form wrinkled surfaces [12]. This occurs because moisture remains in the skin, which remains soft and malleable, and the particles are susceptible to deflation when they cool [30]. Therefore, optimisation of the spray-drying inlet and outlet temperatures appears to be needed to improve the surface characteristics of the powdered particles produced in the current study. It is desirable to obtain smooth spherical particles to maximize the stability of the powder, especially if it is going to be used as a controlled-release agent [31].

The moisture content, water activity, colour, bulk density, WSI and WAI of the powder (Table 6) prepared using the optimal formulation (X_1_ = 35%, X_2_ = 1.5:1 g/g) were also determined because they are important physical properties for indicating the quality and stability of powders [13].

Deterioration of powders can occur when the moisture content level and the water activity are above the critical values of 6% and 0.6, respectively, because microbiological growth and degradation-causing chemical reactions can occur under these conditions [13,32]. Table 6 shows that the moisture content and the water activity were below their critical values for the aqueous extract bitter melon encapsulated powder and therefore, it was likely to be microbiologically stable during storage. These results were similar to findings for other spray dried powders, including for black mulberry juice [33], sumac extract [34] and watermelon [35]. However, the stability of the bitter melon powder during long term storage conditions is yet to be investigated.

The bulk density of the encapsulated bitter melon powder was 0.5 g/mL (Table 6), which is similar to that obtained for encapsulated black mulberry juice [33] and flaxseed oil [15] prepared using MD/GA. It is desirable for a powder to have a high bulk density as it will require less volume when packaged [25]. In addition, a powder with a high bulk density usually has less empty space between particles when it is packed and, therefore, less air occupies these spaces, which can help to prevent oxidation and increase the stability of the powder [36,37].

**Table 6:** The physical properties of the encapsulated powder prepared with the optimal formulation, 35% (w/w) maltodextrin and gum Arabic (MD:GA, 1:1) with a ratio of aqueous bitter melon extract to MD:GA of 1.5:1 g/g.

Physical Properties		
Moisture content (%)		2.82 ± 0.24
Water activity		0.33 ± 0.01
Colour	Lightness	92.41 ± 0.51
	Chroma	7.82 ± 0.19
	Hue	94.79 ± 0.38
Bulk density (g/mL)		0.50 ± 0.04
Water solubility index (%)		93.20 ± 0.36
Water absorption index (%)		1.63 ± 0.03

The results in Table 6 also show that the water solubility of the encapsulated bitter melon powder was high (Table 6). The WSI was 93% and was comparable to the WSI for encapsulated powders prepared for red pitaya peel [38] and acerola pomace extract [39]. The high solubility of the bitter melon powder may be attributed to the fact that MD and GA have superior water solubility [35]. In general, a high WSI is important for the utilisation of powders in the food and pharmaceutical industries because they can be incorporated easily and evenly distributed into products [40].

A low WAI (1.6%) was also obtained for the encapsulated bitter melon extract (Table 6). This meant that its ability to absorb water was low and less likely to be affected by humidity and, therefore, more stable when stored under such conditions [41].

Therefore, because of its low moisture content, low water activity, high bulk density, high WSI and low WAI, it can be concluded that the encapsulated aqueous bitter melon extract powder obtained in this study possessed the desirable characteristics of a high quality powder. Nonetheless, further investigation on controlled release properties of the encapsulated bitter melon extract is needed in order to fully understand how the bioactive compounds release when reconstituted with water.

Furthermore, encapsulation of an aqueous bitter melon extract by spray-drying is highly recommended and the resulting products can be added as fortificants in food products as functional foods and/or as therapeutic agents in the pharmaceutical industry. However, further sensory analysis and evaluation of safety and toxicity tests *in vivo* and human studies are needed prior to commercialisation of the products.

## 4. Conclusions

The formulation for encapsulating an aqueous bitter melon extract by spray-drying was successfully optimised using the RSM with CCD. Second-order polynomial models were generated and found to be statistically adequate to describe and predict the responses for the process yield (*R*^2^ = 0.86) and the retentions of TSC (*R*^2^ = 0.87), TPC (*R*^2^ = 0.98), TFC (*R*^2^ = 0.97) and TAA (*R*^2^ = 0.98).

Using these models, the optimal formulation was predicted and validated to be a concentration (X_1_) of 35% (w/w) for the encapsulating agents’ stock solution and a ratio (X_2_) of 1.5:1 g/g of the aqueous bitter melon extract to the encapsulating agent solution. The process yield and the retentions of TSC, TPC, TFC and TAA were predicted to be 66.2% ± 9.4%, 79.5% ± 8.4%, 92.3% ± 7.3%, 80.7% ± 7.3% and 85.7% ± 7.2%, respectively.

The quality of the encapsulated bitter melon powder prepared using the optimal formulation was high due to its small spherical particle sizes, low moisture content, low water activity, high bulk density, high WSI and low WAI. Therefore, the aqueous bitter melon extract was well encapsulated into a powder using MD/GA and spray-drying.
